# Nuclear Medicine in Diagnosis of Prosthetic Valve Endocarditis: An Update

**DOI:** 10.1155/2015/127325

**Published:** 2015-01-28

**Authors:** Maria Musso, Nicola Petrosillo

**Affiliations:** National Institute for Infectious Diseases “L. Spallanzani”, IRCCS, Via Portuense 292, 00149 Rome, Italy

## Abstract

Over the past decades cardiovascular disease management has been substantially improved by the increasing introduction of medical devices as prosthetic valves. The yearly rate of infective endocarditis (IE) in patient with a prosthetic valve is approximately 3 cases per 1,000 patients. The fatality rate of prosthetic valve endocarditis (PVE) remains stable over the years, in part due to the aging of the population. The diagnostic value of echocardiography in diagnosis is operator-dependent and its sensitivity can decrease in presence of intracardiac devices and valvular prosthesis. The modified Duke criteria are considered the gold standard for diagnosing IE; their sensibility is 80%, but in clinical practice their diagnostic accuracy in PVE is lower, resulting inconclusively in nearly 30% of cases. In the last years, these new imaging modalities have gained an increasing attention because they make it possible to diagnose an IE earlier than the structural alterations occurring. Several studies have been conducted in order to assess the diagnostic accuracy of various nuclear medicine techniques in diagnosis of PVE. We performed a review of the literature to assess the available evidence on the role of nuclear medicine techniques in the diagnosis of PVE.

## 1. Introduction

Endovascular infections, including infective endocarditis (IE) and prosthetic vascular infections, are uncommon pathologies associated with a poor prognosis. Over the past decades cardiovascular disease management has been substantially improved by the increasing introduction of medical devices as mechanical or biologic heart valves, pacemakers, defibrillators, coronary artery stents, and artificial arteries.

The patients' age has increased over the last years, leading to an increase in the number of cardiovascular surgical interventions and also in the infective risk of patients. Between 1990 and 1999 incidence of prosthetic valve endocarditis (PVE) has increased 50% [1, 2]; patients with heart valve prosthesis are exposed to a yearly rate of approximately 3 cases of IE per 1,000 patients [3].

These changes in the epidemiology also explain why, despite important improvements in IE management, the fatality rate of PVE has not decreased in the last forty years, with in-hospital fatality rate of about 20% [4].

PVE are mostly healthcare associated and are distinct in early onset (EO) and late onset (LO), according to the time spent from the intervention to the onset of symptoms; usually a cutoff of one year is adopted [2]. Rates of infection in PVE range from 1–6% to 15%, with higher rates in revision surgery [5].

PVE diagnosis may still be problematic. Imaging investigations traditionally used for diagnosis of PVE are transthoracic echocardiography (TTE) and transesophageal echocardiography (TEE), with a sensitivity of 70% and 90%, respectively [3]; however, the diagnostic value of echocardiography in IE diagnosis is operator-dependent and its sensitivity can decrease in presence of intracardiac devices, valvular prosthesis, severe preexisting lesions, and very small or no vegetation [4].

Over the past years nuclear medicine procedures gave an important contribution to the diagnostic assessment of endovascular infections, through the evaluation of the metabolic activity of the prosthetic material presenting morphological alterations or before development of structural changes.

We performed a review of the literature to summarize the available evidence on the role of nuclear medicine techniques in the diagnosis of PVE.

## 2. Methods

Published papers from January 1980 to June 2014, represented by clinical studies, reviews, and case reports reporting data about use of nuclear medicine techniques to investigate prosthetic valve infections, were found through computerized literature searches using MEDLINE (National Library of Medicine, Bethesda, MD) and by reviewing the references of retrieved articles. Index search terms included the Medical Subjects Heading “nuclear medicine,” “prosthetic,” “valve,” and “endocarditis.” Only articles written in English, German, Spanish, and Italian were included.

Infective endocarditis in patients with an implanted cardiac device and vascular graft was out of the scope of this paper.

## 3. Results and Discussion

### 3.1. Prosthetic Valve Endocarditis Diagnosis

#### 3.1.1. Current Practice in the Management of Endocarditis

The epidemiological characteristics of patients with IE differ between developed and developing countries; in the latter case the patient is younger and is affected by predisposing diseases, such as congenital heart disease or rheumatic fever. In developed countries medical progress has caused an increase in the age of patients with endocarditis and in the incidence of health care associated endocarditis, including PVE [4].

Infection of the cardiac prosthetic valve can occur by contamination at the time of its placement or as a result of hematogenous dissemination or by contiguous infection [2].* Staphylococcus aureus* is the most common cause of PVE, followed by coagulase-negative staphylococci;* Streptococcus viridans* group,* Enterococcus* spp., and yeasts are more common in LO than in EO PVE. Culture-negative endocarditis represents approximately 13% of PVE, with reported rates of 31% [4] and they are more frequent in LO than in EO PVE. Chronic haemodialysis, diabetes, and intravascular devices, including cardiac prosthetic valve, are all predisposing factors to* S. aureus* endocarditis [24].

From a clinical point of view, the presentation of PVE does not substantially differ from the other forms of IE. Indeed, its nonspecific clinical presentation makes IE a cause of fever of unknown origin, with a high clinical suspicion needed to make diagnosis.

The modified Duke criteria are considered the gold standard for diagnosing IE [6] (Tables 1, 2, and 3). In epidemiological studies the sensitivity of Duke criteria is 80%, but in clinical practice their diagnostic accuracy is lower, especially in PVE; prosthetic material in fact compromises the sensitivity of echocardiography that results inconclusively in nearly 30% of cases [4].

PVE represents up to 30% of IE in developed countries, and the in-hospital mortality in patients with PVE can reach 47%, especially in* S. aureus* bacteremia; these data confirm that it is important to increase the ability to early diagnose this clinical condition [8].

#### 3.1.2. The Contribution of Nuclear Medicine in the Diagnosis of PVE

Several nuclear medicine techniques, characterized by different kind of tracers and technologies, have been implemented in order to offer precious information about the metabolic activity of infected tissue. In the last years, these new imaging modalities have gained an increasing attention because they make it possible to diagnose an IE earlier than the structural alterations occurring.

Since the eighties to nowadays several papers have reported the experiences of nuclear medicine (NM) specialists in utilization of NM imaging modalities for PVE diagnosis. Our research provided 29 articles; seven were excluded because they were inadequate to our purpose.

Table 4 summarizes all techniques mentioned in papers reviewed [7–23]; we did not include 6 commentary articles to the prospective study of Saby et al. that we have already included in Table 4 [25–30].

Ten articles reported experiences regarding labeled leukocytes scintigraphy in PVE diagnosis [13–23]; in some of them this technique was associated with single photon emission computed tomography (SPECT) acquisition of images [16–19], because SPECT can provide accurate morphologic details.

Six papers regard ^18^F-fluorodeoxyglucose positron emission tomography in association with computed tomography (^18^F-FDG PET/CT) or with computed tomography angiography (CTA) in the diagnostic assessment of PVE or perivalvular infective complications in patient affected by PVE [12–21].

*(1)* ^*18*^ *F-FDG PET/CT*. ^18^F-FDG PET/CT combines the assessment of metabolic activity of tissues (namely, the glycolytic activity) with the spatial resolution of CT.

A review published in 2013 has extensively collected the available data, mainly from case reports and case series, on the efficacy and usefulness of this technique and has stressed that there is currently no evidence from case-control studies comparing PET/CT with other diagnostic tools currently available [8].

To date there has been only one published prospective study that aims to investigate the diagnostic power of this technique [7]. Seventy-two patients with suspected PVE, initially assessed by using the modified Duke criteria, underwent PET/CT. Although the diagnosis of endocarditis was not always confirmed by histological examination of the prosthetic valve, the authors point out that all those cases of endocarditis undergoing surgery and confirmed by pathologists were identified by ^18^F-FDG PET/CT [7].

Sensitivity and specificity reported in this study (73% and 80%, resp.) did not differ greatly from the values found in a recent study on the use of ^18^F-FDG PET/CT for the diagnosis of endocarditis on pacemakers and implantable defibrillators where sensitivity and specificity were 63% and 86, respectively [31].

However, this study provides interesting data on the possible inclusion of ^18^F-FDG PET/CT among the major criteria of Duke, as already proposed by Millar et al. [7, 8] (Table 2). In fact, the sensitivity of these “new” modified Duke criteria rises up to 97%, which would increase the rate of the so-called “defined” endocarditis [7].

New diagnostic algorithms have been proposed in order to ensure an early diagnosis of endocarditis in those cases in which the Duke criteria are inconclusive; this happens in case of culture-negative endocarditis or in those cases in which transthoracic echocardiography (TTE) and transesophageal echocardiography (TEE) do not visualize lesions of the valve [7, 8].

In this regard, in a case report, Gouriet et al. describe the representative contribution of PET/CT to the diagnosis of PVE in case of “possible endocarditis,” according to the Duke criteria. In this study, PET/CT was performed in course of antibiotic therapy for a knee prostheses infection, after 3 TEEs repeated 7 days apart from each other because of a strong suspicion of IE. The new findings of mitral regurgitation and prolapse of one of the mitral cusps at the third TEE have led to the diagnosis of possible endocarditis; the PET TC subsequently performed made it possible to diagnose a definitive PVE [9].

New imaging modalities, as computed tomography angiography (CTA), have been proposed as a supplement to PET, as Tanis et al. reported in their case series [12]. The authors describe 7 cases of PVE, where the integration of the two techniques allowed diagnosing PVE unidentified by TEE, and periannular complication, that is, mycotic aneurism of coronary arteries and myocardial abscesses [12].

There are still some controversies about various issues related to the PET/CT methodology.

First, the resolving power of this technique permits the detection of lesions of 5 mm and over. However, according to some authors, this is not a limit to the diagnostic power as even smaller lesions, if there is a sufficient uptake, can be displayed. In this regard, Bertagna and colleagues observed that the standard protocol that is used in most nuclear medicine centers is the oncological one that consists of imaging acquisition performed 1 hour after glucose injection [26]. Therefore, they suggest acquiring images even at 2-3 hours after the injection of glucose in order to detect uptake by monocytes and macrophages at the level of the lesion and to increase the sensitivity of the PET/CT methodology [26].

Moreover, there is concern about the specificity of the method in the early period after prosthesis placement. A functional glucose uptake persists in fact from 1 month up to 1 year after surgery. This variability between individuals is influenced by various factors, such as age, clinical status, and overall cardiac condition of the patient. Some authors have also expressed concern about the lack of controls in the prospective study published by Saby et al. [7], because, in order to correctly interpret the values of uptake and the validity of the exam, it is necessary to compare with a normal pattern of glucose uptake, in the absence of infection [30].

*(2) Labeled Leukocytes Scintigraphy*. ^111^Indium is one of the first radiopharmaceuticals used in detecting cardiovascular infections (Table 4) [12–14]. Articles collected [12–14] describe scintigraphy with ^111^Indium employed in diagnosing prosthetic aortic perivalvular abscesses that is a severe endocarditis complication undetectable with traditional radiological tools. This complication is common; it complicates PVE in approximately 50% of cases and needs an urgent surgical approach [20].

^111^Indium seems more efficacious to detect perivalvular complications than valvular surface infections, but its time-consuming images' acquisition limits its utilization.

^67^Gallium citrate has been used more often than ^111^Indium as tracer for scintigraphy in cardiovascular infection field, and it has always been associated with SPECT views [16–19]. One of the articles regarding ^67^Gallium citrate reports its capability to detect endocarditis in absence of any morphological alteration at echocardiography [18].

A retrospective study assessed the value of 99mTc-HMPAO- (hexamethylpropylene amine oxime-) WBC (white blood cells) scintigraphy including SPECT/CT acquisitions in a series of 131 consecutive patients with suspected IE, 68% of whom with prosthetic valves [20].

99mTc-HMPAO-WBC scintigraphy results were correlated with transthoracic or transesophageal echocardiography, blood cultures, and the Duke criteria. ^99m^Technetium-HMPAO-SPECT/CT was positive in all cases of IE in which at least one major Duke criterion was present; moreover, it was positive in IE cases, ascertained by microbiological or clinical diagnosis, with a negative echocardiography [20].

^99m^Technetium labelled monoclonal anti-human granulocytes antibodies (Anti-G mAb) may be employed to label leukocytes in vivo to detect inflammation and infection, but literature regarding its contribution to PVE diagnosis is scarce. Our literature search provided two papers reporting, respectively, a case report and a small prospective case-control study [22, 23]. In this latter article sensitivity and specificity of scintigraphy, in combination with echocardiography, were 100% and 80%, respectively, but the clinical sample was limited, that is, 18 cases and 20 controls [23]. Currently there are no sufficient evidences to suggest this technique as a reliable tool in PVE diagnosis.

#### 3.1.3. The Contribution of Nuclear Medicine to the Diagnosis of Complications in IE, Including PVE

Complications of PVE are rare but worrying because they often require an urgent surgical intervention. They include septic embolism, perivalvular infection, myocardial abscesses, and prosthetic valve dehiscence.

In this regard a retrospective study provided interesting data about the usefulness of ^99m^Technetium-HMPAO-SPECT/CT in assessment of cardiac surgery indications in patients with a suspect PVE [21].

With this technique the authors diagnosed extensive perivalvular infections and abscesses in patients with confirmed endocarditis and no perivalvular extension of the infection detected by TEE; surgery confirmed the presence of an abscess in those patients with positive scintigraphy who underwent surgical intervention, with a positive predictive value for the presence of an abscess of 100% [21].

It is well known that nuclear medicine contributes to the diagnosis of septic metastatic foci in cases of systemic infections, as reported by Vos et al. in a prospective study regarding 115 patients affected by bacteremia [32].

Embolization and/or metastatic infection occurs in more than half of patients with IE and most commonly involves the spleen, the kidney, the liver, and the iliac or mesenteric arteries [33].

Embolic events have been reported in 22–43% of patients with IE, mainly in the first 2 weeks after initiation of antibiotic therapy. A misdiagnosed septic embolism can cause a subsequent seeding of the infection at the metastatic site, a persistent bacteremia, and colonization of newly placed prosthetic valve [34].

Metastatic infection can occur with different form, for example, septic arthritis, spondylodiscitis, pericarditis, and abscess [33, 35]. Their presence influences the appropriate choice of antibiotic therapy and surgical management, the duration of followup, and the outcome of IE [34]. The contribution of ^18^F-FDG PET/CT to the early diagnosis of embolism and metastatic infection in patients with IE has been reported in recent prospective studies [34, 36]; peripheral embolism and/or metastatic infection were detected in 44% of IE episodes, 40% of which were PVE [34].

Moreover, PET/CT detected unexpected extracardiac sites of infection, that is, asymptomatic cases, in 24%–28% of IE [34, 36].

Finally, a retrospective study by Erba et al. on the ^99m^Technetium-HMPAO-SPECT/CT contribution to the assessment of suspected IE reported the usefulness of this technique for the identification of septic emboli, detected in 41% of patients, even in the absence of the typical echocardiographic predictors of systemic embolism [20].

## 4. Conclusions

The early diagnosis of PVE represents a challenge for clinicians involved in its management, including infectious diseases specialist, cardiologist, cardiosurgeons, and imaging physicians, because a delay in antibiotic therapy and cardiac surgery has negative effects on clinical outcomes.

Promising NM techniques, as ^18^F-FDG PET/CT and ^99m^Technetium-HMPAO-SPECT/CT, have been demonstrated to be effective imaging options in the management of PVE. PET has been proposed as a method to increase the sensitivity of the Duke criteria for PVE, and ^99m^Technetium-HMPAO-SPECT/CT is a promising technique that requires further studies. Finally, NM techniques can identify important complications in the course of PVE, including perivalvular abscesses and metastatic embolization, even in asymptomatic patients.

## Figures and Tables

**Table 1:** Modified Duke criteria for the diagnosis of prosthetic valve endocarditis (PVE) [6].

Definite PVE	
*Clinical criteria *	
(1) 2 major criteria	
Or	
(2) 1 major criterion and 3 minor criteria	
Or	
(3) 5 minor criteria	
*Pathological criteria *	
(1) Microorganisms demonstrated by culture or histological examination of a vegetation, a vegetation that has embolized	
Or	
(2) An intracardiac abscess specimen or pathological lesions; vegetation or intracardiac abscess confirmed by histological examination showing active endocarditis	

Possible PVE	
(1) 1 major criterion and 1 minor criterion	
Or	
(2) 3 minor criteria	

Rejected PVE	
(1) Firm alternate diagnosis explaining evidence of infective endocarditis	
Or	
(2) Resolution of infective endocarditis syndrome with antibiotic therapy for ≤4 days	
Or	
(3) No pathological evidence of infective endocarditis at surgery or autopsy, with antibiotic therapy for ≤4 days Or	
(4) Does not meet criteria for possible infective endocarditis, as above	

**Table 2:** Major criteria according to Li et al. [6] and new major criteria according to Saby et al. [7] for the diagnosis of prosthetic valve endocarditis (PVE).

Li et al. [6]	Saby et al. [7]
Blood culture positive for infective endocarditis (i) Typical microorganisms consistent with infective endocarditis from 2 separate blood cultures. *Viridans streptococci, Streptococcus bovis, HACEK group, Staphylococcus aureus,or community-acquired enterococci, in the absence of a primary focus* (ii) Microorganisms consistent with infective endocarditis from persistently positive blood cultures, defined as follows. *≥2 positive cultures of blood samples drawn >*12 h* apart or all of 3 or a majority of ≥4 separate cultures of blood (with first and last sample drawn at least *1 h* apart)* (iii) Single positive blood culture for *Coxiella burnetii* or antiphase I IgG antibody titer >1 : 800 Evidence of endocardial involvement (i) Echocardiogram (TTE and/or TEE) positive for infective endocarditis defined as follows. *Oscillating intracardiac mass on valve or supporting structures, in the path of regurgitant jets, or on implanted material in the absence of an alternative anatomic explanation or* *abscess or* *new partial dehiscence of prosthetic valve* (ii) New valvular regurgitation (worsening or changing of preexisting murmur not sufficient).	Duke major criteria and positive ^18^F-FDG PET/CT: abnormal FDG uptake at the site of prosthetic valve

18F-FDG: 18F-fluorodeoxyglucose; HACEK: *Haemophilus* species, *Actinobacillus actinomycetemcomitans*, *Cardiobacterium hominis*, *Eikenella corrodens*, and *Kingella* species; IgG: immunoglobulin G; PET/CT: positron emission tomography/computed tomography; TEE: transesophageal echocardiography; TTE: transthoracic echocardiography.

**Table 3:** Minor criteria for the diagnosis of prosthetic valve endocarditis (PVE) [6].

(i) Predisposition, predisposing heart condition, or injection drug use (ii) Fever, temperature ≥38°C (iii) Vascular phenomena: major arterial emboli, septic pulmonary infarcts, mycotic aneurysm, intracranial hemorrhage, and conjunctival hemorrhages (iv) Janeway lesions (v) Immunological phenomena: glomerulonephritis, Osler nodes, Roth spots, and rheumatoid factor (vi) Microbiological evidence: positive blood culture but does not meet a major criterion as noted above or serological evidence of active infection with organism consistent with infective endocarditis	

**Table 4:** Nuclear medicine (NM) in the diagnosis of prosthetic valve endocarditis (PVE): review of the literature.

NM imaging modalities	Number of articles/number of patients	Type of paper/reference/year
^ 18^F-FDG-PET/TC	1/not valuable	Review/[8]/2013
	1/72	Prospective study/[7]/2013
	2/2	Case report/[9, 10]/2013, 2009
	1/6	Case series/[11]/2014

CT angiography and ^18^F-FDG-PET	1/7	Case series/[12]/2013

^ 111^Indium labeled leukocytes scintigraphy	2/2	Case report/[13, 14]/1990, 1988
	1/2	Case series/[15]/1989

^ 67^Gallium citrate-SPECT and SPECT/TC	4/4	Case report/[16–19]/2005, 2009, 2002, 1991

^ 99m^Technetium-HMPAO-WBC-SPECT/TC	1/131	Prospective study/[20]/2012
	1/42	Prospective study/[21]/2013

^ 99m^Technetium anti-G mAb-SPECT	1/1	Case report/[22]/1991
	1/38	Prospective study/[23]/1995

FDG: fluorodeoxyglucose; PET/TC: positron emission tomography/computed tomography; CT: computed tomography; SPECT/TC: single photon emission computed tomography/computed tomography; HMPAO: hexamethylpropylene amine oxime; WBC: white blood cells; anti-G mAb: anti-human granulocytes antibodies.

## References

[1] Cabell C. H., Heidenreich P. A., Chu V. H. (2004). Increasing rates of cardiac device infections among Medicare beneficiaries: 1990–1999. *American Heart Journal*.

[2] Baddour L. M., Wilson W. R., Livingstone E. C. (2005). Infections of prosthetic valves and other cardiovascular devices. *Mandell, Douglas and Benett's Principles and Practice of Infectious Diseases*.

[3] Iung B., Erba P. A., Petrosillo N., Lazzeri E. (2014). Common diagnostic flowcharts in infective endocarditis. *The Quarterly Journal of Nuclear Medicine and Molecular Imaging*.

[4] Thuny F., Grisoli D., Collart F., Habib G., Raoult D. (2012). Management of infective endocarditis: challenges and perspectives. *The Lancet*.

[5] Petrosillo N., Signore A., Quintero A. M. (2014). Epidemiology of infections in the new century. *Diagnostic Imaging of Infections and Inflammatory Diseases: A Multidisciplinary Approach*.

[6] Li J. S., Sexton D. J., Mick N. (2000). Proposed modifications to the Duke criteria for the diagnosis of infective endocarditis. *Clinical Infectious Diseases*.

[7] Saby L., Laas O., Habib G. (2013). Positron emission tomography/computed tomography for diagnosis of prosthetic valve endocarditis: increased valvular ^18^F-fluorodeoxyglucose uptake as a novel major criterion. *Journal of the American College of Cardiology*.

[8] Millar B. C., Prendergast B. D., Alavi A., Moore J. E. (2013). ^18^FDG-positron emission tomography (PET) has a role to play in the diagnosis and therapy of infective endocarditis and cardiac device infection. *International Journal of Cardiology*.

[9] Gouriet F., Bayle S., le Dolley Y. (2013). Infectious endocarditis detected by PET/CT in a patient with a prosthetic knee infection: Case report and review of the literature. *Scandinavian Journal of Infectious Diseases*.

[10] Moghadam-Kia S., Nawaz A., Millar B. C. (2009). Imaging with ^18^F-FDG-PET in infective endocarditis: promising role in difficult diagnosis and treatment monitoring. *Hellenic Journal of Nuclear Medicine*.

[11] Bartoletti M., Tumietto F., Fasulo G. (2014). Combined computed tomography and fluorodeoxyglucose positron emission tomography in the diagnosis of prosthetic valve endocarditis: a case series. *BMC Research Notes*.

[12] Tanis W., Scholtens A., Habets J. (2013). CT angiography and ^18^F-FDG-PET fusion imaging for prosthetic heart valve endocarditis. *JACC: Cardiovascular Imaging*.

[13] Purnell G. L., Walker C. W., Allison J. W., Dalrymple G. V. (1990). Indium-111 leukocyte localization in infected prosthetic graft. *Clinical Nuclear Medicine*.

[14] Oates E., Sarno R. C. (1988). Detection of a prosthetic aortic valvular abscess with indium-111-labeled leukocytes. *Chest*.

[15] Cerqueira M. D., Jacobson A. F. (1989). Indium-111 leukocyte scintigraphic detection of myocardial abscess formation in patients with endocarditis. *Journal of Nuclear Medicine*.

[16] Thomson L. E. J., Goodman M. P., Naqvi T. Z. (2005). Aortic root infection in a prosthetic valve demonstrated by gallium-67 citrate SPECT. *Clinical Nuclear Medicine*.

[17] Yavari A., Ayoub T., Livieratos L., Raman V., McWilliams E. T. (2009). Diagnosis of prosthetic aortic valve endocarditis with gallium-67 citrate single-photon emission computed tomography/computed tomography hybrid imaging using software registration. *Circulation: Cardiovascular Imaging*.

[18] Pena F. J., Banzo I., Quirce R. (2002). Ga-67 SPECT to detect endocarditis after replacement of an aortic valve. *Clinical Nuclear Medicine*.

[19] O'Brien K., Barnes D., Martin R. H., Rae J. R. (1991). Gallium-SPECT in the detection of prosthetic valve endocarditis and aortic ring abscess. *Journal of Nuclear Medicine*.

[20] Erba P. A., Conti U., Lazzeri E. (2012). Added value of ^99m^Tc-HMPAO-labeled leukocyte SPECT/CT in the characterization and management of patients with infectious endocarditis. *The Journal of Nuclear Medicine*.

[21] Hyafil F., Rouzet F., Lepage L. (2013). Role of radiolabelled leucocyte scintigraphy in patients with a suspicion of prosthetic valve endocarditis and inconclusive echocardiography. *European Heart Journal: Cardiovascular Imaging*.

[22] Bair H. J., Becker W., Volkholz H. J., Wolf F. (1991). 99mTc-labelled anti NCA-95 antibodies in prosthetic heart valve endocarditis. *NuklearMedizin*.

[23] Morguet A. J., Munz D. L., Ivancevic V., Werner G. S., Kreuzer H. (1995). Clinical value of radioimaging using the murine monoclonal antigranulocyte antibody BW 250/183 in the diagnosis of prosthetic valve endocarditis. *Deutsche Medizinische Wochenschrift*.

[24] Liu C., Bayer A., Cosgrove S. E. (2011). Clinical practice guidelines by the Infectious Diseases Society of America for the treatment of methicillin-resistant *Staphylococcus aureus* infections in adults and children: executive summary. *Clinical Infectious Diseases*.

[25] Dilsizian V., Achenbach S., Narula J. (2013). Adding or selecting imaging modalities for incremental diagnosis: a case study of ^18^FDG PET/CT in prosthetic valve endocarditis. *JACC Cardiovascular Imaging*.

[26] Bertagna F., Giubbini R., Treglia G. (2014). Positron emission tomography/computed tomography for diagnosis of prosthetic valve endocarditis: suggestions to increase diagnostic accuracy. *Journal of the American College of Cardiology*.

[27] Sharma A. (2013). Positron emission tomography/computed tomography for diagnosis of prosthetic valve endocarditis. *Journal of the American College of Cardiology*.

[28] Thuny F., Saby L., Tessonier L., Cammilleri S., Raoult D., Habib G. (2013). Reply: positron emission tomography/computed tomography for diagnosis of prosthetic valve endocarditis. *Journal of the American College of Cardiology*.

[29] Thuny F., Lass O., Saby L. (2014). Reply: positron emission tomography/computed tomography for diagnosis of prosthetic valve endocarditis. *Journal of the American College of Cardiology*.

[30] Tanis W., Scholtens A., Habets J. (2014). Positron emission tomography/computed tomography for diagnosis of prosthetic valve endocarditis: increased valvular ^18^F-fluorodeoxyglucose uptake as a novel major criterion. *Journal of the American College of Cardiology*.

[31] Graziosi M., Nanni C., Lorenzini M. (2014). Role of ^18^F-FDG PET/CT in the diagnosis of infective endocarditis in patients with an implanted cardiac device: a prospective study. *European Journal of Nuclear Medicine and Molecular Imaging*.

[32] Vos F. J., Bleeker-Rovers C. P., Sturm P. D. (2010). ^18^F-FDG PET/CT for detection of metastatic infection in gram-positive bacteremia. *The Journal of Nuclear Medicine*.

[33] Mylonakis E., Calderwood S. B. (2001). Infective endocarditis in adults. *The New England Journal of Medicine*.

[34] van Riet J., Hill E. E., Gheysens O. (2010). ^18^F-FDG PET/CT for early detection of embolism and metastatic infection in patients with infective endocarditis. *European Journal of Nuclear Medicine and Molecular Imaging*.

[35] Cone L. A., Hirschberg J., Lopez C. (2008). Infective endocarditis associated with spondylodiscitis and frequent secondary epidural abscess. *Surgical Neurology*.

[36] Bonfiglioli R., Nanni C., Morigi J. J. (2013). ^18^F-FDG PET/CT diagnosis of unexpected extracardiac septic embolisms in patients with suspected cardiac endocarditis. *European Journal of Nuclear Medicine and Molecular Imaging*.

